# Malignant Remittent Fever

**Published:** 1880-03-20

**Authors:** D. L. Mackenzie


### *CASE OF MALIGNANT REMITTENT FEVER.*

BY D. L. MACKENZIE, L.R.C.P., ETC.

The following case may be interesting in view of the late epidemic of yellow fever on the banks of the Mississippi, and its present prevalence at the French settlements of Dacca and Goree on this coast. It is a typical case of the severest form of malarious fever, of rare occurrence, and although very similar to yellow fever, can, I think, be readily distinguished from it. It is interesting, also, on account of the proximity of yellow fever to these parts, and because an epidemic of this disease usually appears on the West Coast of Africa every sixth or seventh year, as the epidemics of 1860, 1866 and 1873 testify, when fully one-half of the European residents succumbed to its ravages. In 1872 the epidemic, which in the following year visited Calabar, Bonny, and the other large rivers in this part of the coast, commenced in Senegambia and Sierra Leone, and it is feared that ere long the same track may be taken by the deadly scourge. So far, the settlements in this neighborhood have, with the exception of Lagos, been comparatively healthy; but in the latter place, during the month of April, May, June and July, nearly one-third of its entire European population died from fever and dysentery alone.

Thomas C——, aged 27, a Scotchman by birth, of delicate constitution, had, after considerable exposure to fatigue and malarious influences, complained of fever of an intermittent type for five days, accompanied by constipation of bowels and sleeplessness, which, on October 7th, culminated in a rigor of more than usual severity. He had frequently suffered from malarious fevers previously. Bowels had been freely opened by means of a simple enema. On visiting him I found the cold stage had given place to the hot. He was suffering from high fever. The pulse was rapid (130), weak and compressible; temperature 105°; skin hot and dry, of a bright-yellow hue all over the body. There was great precordial oppression, with nausea and vomiting of blood. The urine also contained blood, and the same material was passed in large quantity by the bowels. The liver and spleen were tender on heavy pressure, but not painful; they were slightly enlarged, probably no more so than in an ordinary case of uncomplicated remittent. There was no headache, but great restlessness prevailed; tongue dry and covered with a brown fur; thirst intense. Seeing that hemorrhage was the most serious symptom, I administered gallic acid in fifteen-grain doses, repeated every four hours; cold was applied to the head, and enemata of beef-tea and port-wine were also administered; champagne and occasionally diluted lime-water were allowed.

*Evening.* All bleeding stopped. Patient very hot and restless; vomiting of bile very distressing; bowels repeatedly open; stools bilious; nausea and oppression at the epigastrium very severe; no pain complained of anywhere except in the small of the back; thirst intense; effervescing draugths, with chloroform were ordered to be given at short intervals. A sinapism was applied to the epigastrium, and the injections to be given every hour.

*October* 10th.—Patient much exhausted. Pulse very weak and rapid; temperature nearly 106°; tongue dry and brown; vomiting and thirst urgent; urine dark-colored, containing blood and bile; stools and matters vomited also containing large quantities of bile; the yellow tinge of skin if anything brighter than yesterday. Brandy was substituted for port wine in the enemata, and fifteen grains of sulphate of quinine were ordered to be added to three of these during the course of the day.

11th.—Patient much the same. Restlessness, with vomiting and dry hot skin, as severe as ever; temperature and pulse unreduced; secretions much the same, excepting the urine, which contained no blood or albumen. Hydrocyanic acid, with bismuth, were substituted for chloroform in the effervescing draughts, and as no sleep had been obtained, a solution containing half a grain of acetate of morphia was subcutaneously injected. The enemata were well retained.

12th.—Slight improvement observable, although the vomiting and uneasiness at the epigastrium, with thirst and undiminished fever, still continue. Quietness, if not a little sleep, has been obtained by the effect of the morphia, which was repeated to-day, and again in the evening. Chloroform being more effectual than hydrocyanic acid in the draughts, was again resorted to. The injections with quinine continued.

13th.—The vomiting, now freely bilious, is at times replaced by retching. Pulse 120, of slightly greater strength; temperature 103°. Same treatment, with cold sponging, pursued.

14th.—Profuse perspiration has at length taken place. The favorable change, being more marked than yesterday, is now visible to patient's attendants. The skin is more natural in color, and retching less distressing. Cinchonism is present. Pulse 100; temperature 100°.

15th.—To day, for the fisrt time, a small quantity of beef-tea has been retained, and considerable improvement in the condition of the patient is noted. Injections, with quinine in slightly diminished quantity, continued.

16th.—Food in the form of beef-tea and chicken-soup has been repeatedly taken yesterday and to-day. Temperature now normal; skin moist, and no sign of a recurrence of the hot stage.

17th.—Injections now limited to one per day, and that more as a vehicle for quinine than for the purposes of nutrition, the stomach being still incapable of receiving irritative substances.

19th.—Convalescence being now fairly established, quinine was omitted. Strength daily on the increase. Very little sleep is, however, yet obtained; so a trial of bromide of potassium was made to day.

21st.—Enemata now discontinued. None but liquid foods, however, yet given. Convalescence appearing to be very tardy, a tonic of iron and quinine was to-day commenced.

24th.—Health is being gradually attained, and a trial of solid food has been to-day made. Sight, which has been hitherto very dim, is now nearly natural.

28th.—Progress being slow, I have to-day ordered my patient to proceed for change to a station some two miles distant.

*November* 4th.—Great benefit has been derived from the change, my patient being now in his normal state of health.

The case speaks for itself. To me the points of difference between it and yellow fever consist in the absence of albumen in the urine after hemorrhage had ceased, the absence of serious cerebral mischief, the absence also of the severe pain in the back so much insisted on by some authors, American and French, as being symptomatic of yellow fever, and the fact of its being so comparatively amenable to quinine. The similarity to yellow fever cannot be doubted. It is the nearest approach to it, excepting the 1873 epidemic, I have seen in nearly nine years' practice in Western Africa, and recovery from such malignant attacks must be rare indeed.—*London Lancet.*

